# Triterpenoid Saponin and Lignan Glycosides from the Traditional Medicine *Elaeagnus angustifolia* Flowers and Their Cytotoxic Activities

**DOI:** 10.3390/molecules25030462

**Published:** 2020-01-22

**Authors:** Jianxin Han, Xiaoyu Chen, Wei Liu, Hao Cui, Tao Yuan

**Affiliations:** 1 Department of Food Science and Nutrition, School of Biosystems Engineering and Food Science, Zhejiang Key Laboratory for Agro-Food Processing, Zhejiang University, Hangzhou 310058, China; jianxin@zju.edu.cn; 2 The Key Laboratory of Plant Resources and Chemistry of Arid Zone, and State Key Laboratory of Xinjiang Indigenous Medicinal Plants Resource Utilization, Xinjiang Technical Institute of Physics and Chemistry, Chinese Academy of Sciences, Urumqi 830011, China; chenxiaoyu16@mails.ucas.edu.cn (X.C.); ucasliuwei@126.com (W.L.); 3 The Laboratory of Effective Substances of Jiangxi Genuine Medicinal Materials, College of Life Sciences, Jiangxi Normal University, Nanchang 330022, China; cuihaoeric@jxnu.edu.cn

**Keywords:** *Elaeagnus angustifolia*, Elaeagnaceae, triterpenoid saponin, lignans, cytotoxicity, A375

## Abstract

A new triterpenoid saponin, named terpengustifol A (**1**), and two new lignan glucosides, phengustifols A and B (**2** and **3**), were isolated from the flowers of *Elaeagnus angustifolia*. Their structures were determined by the extensive analysis of the spectroscopic data (including NMR and HRMS) and ECD calculations. Compound **1** possesses an unusual monoterpene (*Z*)-6-hydroxy-2,6-dimethylocta-2,7-dienoyl unit at C-21. Compounds **2** and **3** are a pair of diastereoisomers, while their aglycones are a pair of enantiomers. Compounds **1** and **2** exhibited moderate cytotoxic activities against A375 cell lines with IC_50_ values at 12.1 and 15.6 μM, respectively. This is firstly reported the triterpenoid saponin and lignans isolated from the *Elaeagnus angustifolia* flowers.

## 1. Introduction

**Figure 1:**
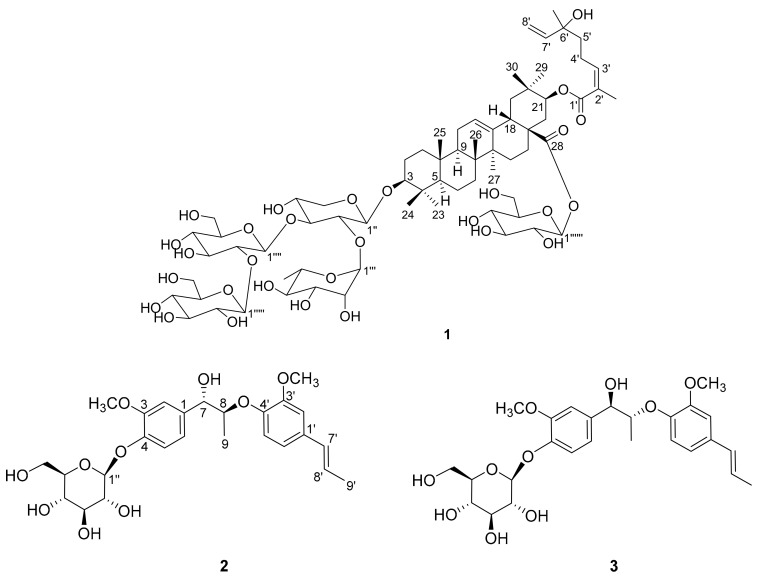
Chemical structures of compounds **1**–**3**.

*Elaeagnus angustifolia* L., commonly called oleaster or Russian olive, is a medicinal plant belonging to the Elaeagnacea family. It is widely distributed from the northern regions of Asia to the Himalayas and Europe [1]. *E. angustifolia* is mainly planted in various provinces of Northwest of China, for wind breaks and sand fixation [2]. The flowers of *E. angustifolia* were traditionally used to treat asthma and tetanus in Iran and China [3,4,5]. In Chinese Uygur medicine, *E. angustifolia* flowers were considered as dry and hot materials, which is mainly used to treat brain disease, thoracalgia and asthma in the clinic. According to the specialists in traditional medicine, *E. angustifolia* flowers are a herbal medication which can stimulate sexuality, especially in young girls and women [6]. Zeinalzadeh et al. reported a randomized clinical trial to compare the effect of *E. angustifolia* flower capsule and sildenafil citrate tablet on sexual interest/arousal disorder [7]. Previous phytochemical investigations of *E. angustifolia* flowers focused on the essential oil and the total flavonoids content, and few studies were conducted on the isolation and identification of compounds present in the flowers [8,9,10]. Our group previously reported the identification of a novel macrocyclic flavonoid glycoside from the flowers of this plant [11]. In continuation of this program, a new triterpenoid saponin (**1**), and two new lignan glucosides (**2** and **3**) (Figure 1 and Supplementary Materials), were further isolated and identified from *E. angustifolia* flowers. Herein, the isolation, structural elucidation, and cytotoxic activities of the compounds, were presented.

## 2. Results and Discussion

Compound **1** was isolated as white powder; its molecular formula was identified as C_69_H_110_O_29_ by the pseudomolecular ion peak at *m*/*z* 1447.6933 [M + HCOO]^-^ (calcd for C_70_H_111_O_31_, 1447.7109) in the HRESIMS spectrum. The ^1^H-NMR spectrum of **1** (Table 1) showed signals for nine tertiary methyl groups at *δ*_H_ 1.81, 1.27, 1.17, 1.04, 1.03, 0.95, 0.90, 0.86, and 0.79 (each 3H, s), a secondary methyl at *δ*_H_ 1.21 (3H, d, *J* = 6.2 Hz), several anomeric protons and olefinic protons signals at *δ*_H_ 4.30–6.00, as well as many sugar moiety protons signals at *δ*_H_ 3.00–4.10. The ^13^C NMR spectrum (with HSQC experiments, Table 1) revealed the presence of 69 carbon resonances including five anomeric carbons (*δ*_C_ 106.2, 105.9, 103.5, 101.3 and 95.9), and two ester carbonyls (*δ*_C_ 176.3 and 169.3). The above-mentioned data was typical for triterpenoid saponin, and the olefinic proton signal at *δ*_H_ 5.30 (1H, t, *J* = 3.3 Hz, H-12) implied that it is an oleanolic-type saponin [12].

Acid hydrolysis of compound **1** yielded xylose, glucose and rhamnose, their configurations were identified as d-configuration for xylose and glucose, and l-configuration for rhamnose by the method gave in the experiment part. Analysis of HSQC, HMQC-TOCSY, and ^1^H-^1^H COSY spectrum clarified the proton and carbon signals of five sugar units (a xylose, three glucoses and a rhamnose) as shown in Table 1. Aside from the signals for the sugar units, 40 other carbon signals were observed in the ^13^C-NMR spectrum. Further analysis of 2D NMR data revealed the presence of an aglycone with 3,21-dihydroxyolean-12-en-28-oic acid skeleton. There are 10 carbon signals remaining except the five sugar units and 3,21-dihydroxyolean-12-en-28-oic acid skeleton.

**Figure 2:**
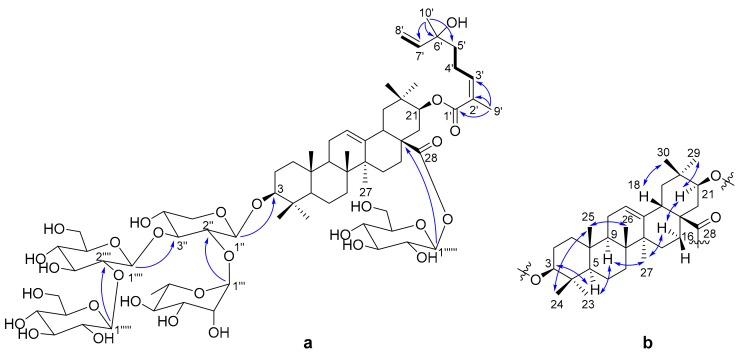
(**a**) Key ^1^H-^1^H COSY (−) and selected HMBC correlations (H→C) of **1**; (**b**) Key NOESY (↔) correlations of **1**.

The ^1^H-^1^H COSY correlations (Figure 2a) of H-3′/H-4′/H-5′ and H-7′/H-8′, and the HMBC correlations (Figure 2a) from H_3_-9′ to C-1′ (*δ*_C_ 169.3), C-2′ (*δ*_C_ 129.0) and C-3′ (*δ*_C_ 144.3); from H_3_-10′ to C-5′ (*δ*_C_ 41.7), C-6′ (*δ*_C_ 73.7) and C-7′ (*δ*_C_ 146.1), indicated the presence of a 6-hydroxy-2,6-dimethyl-octa-2,7-dienoyl monoterpene moiety. Thus, the three units (sugars, triterpenoid and monoterpene) of **1** were determined, and their connections are the next thing to solve. The HMBC correlation from H-1′′ to C-3 (*δ*_C_ 90.0) connected the xylose to C-3. A rhamnose and glucose were linked to positions 2 and 3 of xylose, respectively, by the HMBC correlations (Figure 2a) from H-1′′′ to C-2′′ (*δ*_C_ 75.5) and from H-1′′′′ to C-3′′ (*δ*_C_ 83.2). The HMBC correlation from H-1′′′′′ to C-2′′′′ (*δ*_C_ 83.9) assigned another glucose to position 2 of the glucose connected to xylose. The last glucose was connected to C-28 on the basis of the HMBC correlation from H-1′′′′′′ to C-28 (*δ*_C_ 176.3). Compared the CH-21 (*δ*_H_ 4.81, *δ*_C_ 77.0) chemical shift of **1** to that of known compound machaerinic acid [13], obvious down-field shift indicated the monoterpene moiety was attached to C-21, although the HMBC correlation from H-21 to C-1′ was not observed. The NOESY correlations (Figure 2b): from H-3 to H-5, from H-5 to H-9, from H-9 to H_3_-27, from H_3_-27 to H-16α, from H-16α to H-21, and from H-21 to H_3_-29, indicated they were in the same face. Correspondingly, the NOESY correlations from H_3_-24 to H_3_-25, from H_3_-25 to H_3_-26, and from H-18 to H_3_-30, revealed they took β-orientation. The *Z*-geometry of Δ^2^^′^ was determined by the NOESY correlation between H-3′ and H_3_-9′. Therefore, the structure of compound **1** was elucidated as shown in Figure 1, and it was given the trivial name terpengustifol A.

Compounds **2** and **3** were obtained originally as a mixture as they were showed as one peak in the HPLC chromatography using a reverse phase C_18_ column. The ^1^H-NMR spectrum of the mixture cannot distinguish they are two compounds, while there are some subtle differences of several carbon resonances in the ^13^C-NMR spectrum. Chiral chromatography analysis of the mixture showed that there were two peaks with a ratio around 1:1. Thus, compounds **2** and **3** were separated by chiral chromatography. The molecular formula of compounds **2** and **3** were established as C_26_H_34_O_10_ by their HRESIMS data (*m*/*z* 551.2091 and 551.2086 [M + HCOO]^−^; calcd for C_27_H_35_O_12_, 551.2129). In the ^1^H- NMR spectrum (Table 2) of **2**, the signals for two 1,3,4-trisubstituted aromatic rings [*δ*_H_ 7.15 (1H, d, *J* = 8.3 Hz, H-5), 7.11 (1H, d, *J* = 1.8 Hz, H-2), 6.97 (1H, dd, *J* = 8.3, 1.8 Hz, H-6); 6.98 (1H, d, *J* = 1.9 Hz, H-2′), 6.90 (1H, d, *J* = 8.3 Hz, H-5′), 6.84 (1H, dd, *J* = 8.3, 1.9 Hz, H-6′)], a *trans* double bond signals [*δ*_H_ 6.33 (1H, dd, *J* = 15.7, 1.6 Hz, H-7′), 6.15 (1H, dq, *J* = 15.7, 6.5 Hz, H-8′)], two oxygenated methines [*δ*_H_ 4.70 (1H, d, *J* = 6.1 Hz, H-7), 4.43 (1H, dq, *J* = 6.2, 6.1 Hz, H-8)], a series of proton signals fa or sugar moiety at *δ*_H_ 3.00–5.00, two methoxy groups signals (*δ*_H_ 3.87, 3.85, each 3H, s), and two methyl signals [*δ*_H_ 1.85 (3H, dd, *J* = 6.5, 1.6 Hz, H-9′), 1.08 (3H, d, *J* = 6.2 Hz, H-9)], were observed. The anomeric proton resonated at *δ*_H_ 4.88 (1H, d, *J* = 7.5 Hz, H-1′′) suggested it is in a β-configuration. The ^13^C-NMR and HSQC spectrum of **2** showed 26 carbon resonances, corresponding to four methyls, one methylene, 15 methines (including eight sp^2^ carbons), and six quaternary carbons. In addition to the sugar moiety and two methoxy groups, the remaining 18 carbons suggested that compound **2** is likely a lignan glycoside.

**Figure 3:**
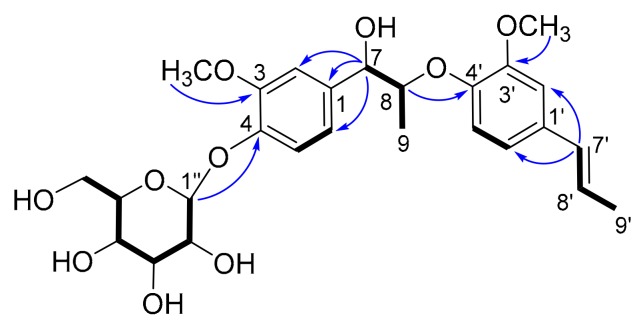
Key ^1^H-^1^H COSY (−) and selected HMBC correlations (H→C) of **2**.

Further analysis of 2D NMR data (^1^H-^1^H COSY, HSQC, HMBC) of **2** established the planar structure. A hexose moiety (C-1′′ to C-6′′) and two subunits (C-7 to C-9 and C-7′ to C-9′) (drawn with bold bond in Figure 3) were established based on the ^1^H–^1^H COSY correlations. The three fragments were connected to the other functional groups by the HMBC correlations (Figure 3). The linkage between C-7 and C-1 was determined by the HMBC correlations from H-7 to C-1, C-2 and C-6, and the linkage between C-8 and C-4′ via ether bond by the HMBC correlations from H-8 to C-4′. The fragment C-7′ to C-9′ was linked to C-1′ by the HMBC correlation from H-7′ to C-2′ and C-6′.

The HMBC correlation from H-1′′ to C-4 indicated the hexose moiety was connected to C-4. Thus, the planar structure of **2** was elucidated as depicted.

**Figure 4:**
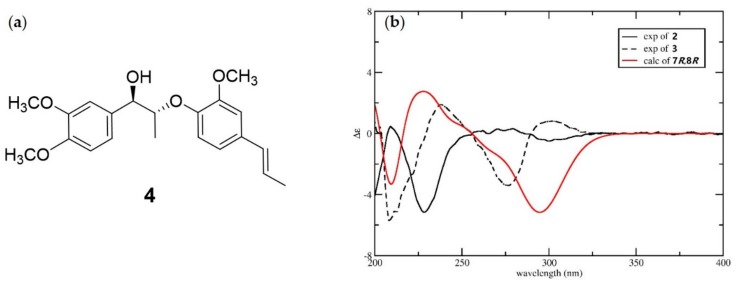
(**a**) Chemical structure of model compound **4**; (**b**) Experimental ECD spectra of **2** and **3**, and calculated ECD spectra for **4**.

Compound **3** had the same planar structure as **2** based on analysis of the NMR data. Both compounds **2** and **3** produced d-glucose after acid hydrolysis, indicating that the difference between them was the stereochemistry of C-7 and C-8. The large coupling constant between H-7 and H-8 (*J* = 6.1 Hz) of compounds **2** and **3** indicated they had the same 7,8-*threo* configuration [14]. Therefore, compounds **2** and **3** are a pair of diastereoisomers, making their aglycones a pair of enantiomers. An ECD calculation was applied to elucidate the absolute configurations of C-7 and C-8. Due to the excessive number of chiral centers of glucose, a model compound **4** (Figure 4a) was selected to simplify the calculation. As shown in the Figure 4b, the calculated ECD of (7*R*,8*R*)-**4** showed a similar Cotton effect as compound **3**, which indicated that compound **3** had a 7*R*,8*R*-configuration. Correspondingly, compound **2** possesses a 7*S*,8*S* configuration. Thus, the structures of compounds **2** and **3** were determined as shown in Figure 1, and they were given the trivial names phengustifol A and B, respectively.

There are many reports about the cytotoxicity of triterpenoid saponins and lignan glucosides against various cell lines [15,16,17,18], thus, the three new compounds obtained in the current study were tested for cytotoxic activity against the A375 human melanoma cell line using the CCK8 method [19]. The results revealed that compounds **1** and **2** exhibited moderate cytotoxic activities, with IC_50_ values at 12.1 and 15.6 μM, respectively, while compound **3** showed weak cytotoxicity with an IC_50_ value of 62.8 μM. The IC_50_ value for the positive control cabazitaxel was 0.11 μM.

## 3. Materials and Methods

### 3.1. General Procedures

UV spectra were measured on a UV-2550 UV-visible spectrophotometer (Shimadzu, Shimane-ken, Japan). IR spectra were recorded on a 380 FT-IR spectrometer (Thermo Nicolet, Waltham, MA, USA). The optical rotations were measured on an AutoPol IV automatic polarimeter (Rudolph Research, Wilmington, MA, USA) at room temperature. 1D and 2D NMR data were recorded on a 600 MHz instrument (Varian, Palo Alto, CA, USA) with TMS as internal standard. HRESIMS data were acquired using a Triple TOF 6600 mass spectrometer (AB Sciex, Framingham, MA, USA). Semi-preparative HPLC separations were performed on a Chromaster system (Hitachi, Tokyo, Japan) consisting of a 5110 pump, 5210 autosampler, 5310 column oven, 5430 diode array detector and a Phenomenex Luna C_18_ column (250 × 10 mm, S-5 μm), all operated using EZChrom Elite software. All solvents were of ACS or HPLC grade, and were obtained from Tansoole (Shanghai, China) and Sigma-Aldrich (St. Louis, MO, USA), respectively. Silica gel (300–400 mesh), C_18_ reverse-phased silica gel (150-200 mesh, Merck, Darmstadt, German), and MCI gel (CHP20P, 75–150 μM, Mitsubishi Chemical Industries Ltd., Tokyo, Japan) were used for column chromatography (CC), and pre-coated silica gel GF254 plates (Qingdao Marine Chemical Plant, Qingdao, China) were used for TLC.

### 3.2. Plant Material

*Elaeagnus angustifolia* flowers were collected from Changji (Xinjiang Province, China) and identified by Prof. Yan Wei (College of Grassland and Environment Sciences, Xinjiang Agricultural University). A voucher specimen (EA-201506) is deposited in the Key Laboratory of Plant Resources and Chemistry of Arid Zone, Xinjiang Technical Institute of Physics and Chemistry, Chinese Academy of Sciences (Xinjiang, China).

### 3.3. Extraction and Isolation

Air-dried ground powder of *E. angustifolia* flowers (1.0 kg) was sequentially extracted with petroleum ether (8 L × 3) and methanol (10 L × 3) by maceration at room temperature (7 days each time) to afford a crude methanol extract. The crude methanol extract was suspended in distilled water and then extracted successively with petroleum ether (PE), ethyl acetate and *n*-butanol. The *n*-butanol fraction (69.2 g) was subjected to a column of MCI gel (MeOH-H_2_O, 10:90 to 100:0, *v*/*v*) to yield six fractions (A-F). Fraction F (3.5 g) was subjected to a C18 reverse-phased silica gel column eluting with step gradient MeOH-H_2_O (30:70 to 60:40, *v*/*v*) to give 12 fractions F1-F12. Fraction F5 (300.0 mg) was separated on Sephadex LH-20 column eluted with MeOH to yield 11 sub-fractions F5a–F5k. Sub-fraction F5e (36.4 mg) was purified by semi-preparative HPLC, eluting with isocratic MeOH-H_2_O (57:43, *v*/*v*, 3 mL/min) to yield the mixture of compounds **2** and **3**. The mixture was then separated on chiral column (CHIRALPAK^®^ AD-H, 10 × 250 mm, 5 μm, Daicel Chiral) eluting with n-hexane/isopropanol (70/30, *v*/*v*) at 3 mL/min flow rate to get compounds **2** (2.4 mg) and **3** (2.2 mg). Fraction F9 (105.0 mg) was subjected to a Sephadex LH-20 column eluted with MeOH to yield eight sub-fractions F9a–F9h. Purification of F9b (36.9 mg) using semi-preparative HPLC eluted with MeOH/H_2_O (70/30 to 80/20 in 30 min, 3mL/min) to yield compound **1** (6.4 mg).

*Terpengustifol A* (**1**): white amorphous powder; ${\left[\alpha \right]}_{D}^{20}$ = +44 (*c* 0.200, MeOH); UV (MeOH) *λ*_max_: 202, 220 nm; IR ν_max_ 3422, 2926, 1685, 1676, 1610, 1437, 1388, 1076, 1018 cm^−1^; ^1^H-NMR and ^13^C-NMR data, see Table 1; HR-ESI-MS: *m/z* 1447.6933 [M + HCOO]^−^ (calcd for C_70_H_111_O_31_, 1447.7109).

*Phengustifol A* (**2**): white amorphous powder; ${\left[\alpha \right]}_{D}^{20}$ = −48 (*c* 0.05, MeOH); UV (MeOH) *λ*_max_: 204, 260 nm; IR ν_max_ 3427, 2921, 1682, 1508, 1456, 1264, 1031, 801 cm^−1^; ^1^H-NMR and ^13^C-NMR data, see Table 2; HR-ESI-MS: *m/z* 551.2091 [M + HCOO]^−^ (calcd for C_27_H_35_O_12_, 551.2129).

*Phengustifol B* (**3**): white amorphous powder; ${\left[\alpha \right]}_{D}^{20}$ = −12 (*c* 0.05, MeOH); UV (MeOH) *λ*_max_: 204, 260 nm; ^1^H-NMR and ^13^C-NMR data, see Table 2; HR-ESI-MS: *m/z* 551.2086 [M + HCOO]^−^ (calcd for C_27_H_35_O_12_, 551.2129).

### 3.4. Acid Hydrolysis of Compounds **1**-**3** and Sugar Analysis

Each compound (1.0 mg) was added to 1 mL HCl (1 N) and refluxed for 2 h. The solution was evaporated under a stream of N_2_ after cooling. Mixture of the residue, anhydrous pyridine solution (0.1 mL) and l-cysteine methyl ester hydrochloride (0.06 N) was heated at 60 °C for half an hour. Removing the solvent, the residue was partitioned between water and cyclohexane. The cyclohexane layer was dried and dissolved in 200 μL acetone for GC analysis.

### 3.5. Cytotoxicity Assay

The Cell Counting Kit 8 (CCK-8) assay was used to evaluate the cytotoxicity of the compounds against A375 cells. The cells were cultured in DMEM with 10% FBS in a humidified incubator with 5% CO_2_ at 37 °C for 24 h. The test compounds and positive control were added to the cultures and incubated for another 24 h. After that, the supernatant was removed, 10 μL of CCK8 (Dojindo, Kumamoto, Japan) reagent was added per well and incubated at 37 °C for 2 h. The samples were then transferred to a microplate reader to measure the optical density at a wavelength of 450 nm. Cabazitaxel was used as positive control.

## 4. Conclusions

In the present work, three new compounds **1**–**3**, including a triterpenoid saponin and two lignan glucosides, were isolated and identified from *Elaeagnus angustifolia* flowers. Compound **1** possesses an unusual (*Z*)-6-hydroxy-2,6-dimethylocta-2,7-dienoyl monoterpene unit at C-21. Compounds **2** and **3** are a pair of diastereoisomers, while their aglycones are a pair of enantiomers. The bioassay indicated that all of the compounds showed somehow cytotoxic activities against A375, compounds **1** and **2** exhibited moderate activities. Interestingly, compound **2** showed stronger cytotoxic activities than that of **3** due to the different absolute configurations of their aglycones.

## Figures and Tables

**Table 1:** ^1^H- and ^13^C-NMR spectroscopic data of **1**^*a*^ (in CD_3_OD).

Genin part	*δ*_H_ (Mult; *J*, Hz)	*δ*_C_	Sugar Part	*δ*_H_ (Mult; *J*, Hz)	*δ*_C_
1	1.60 (m), 0.96 (m)	40.4	Xyl (C-3)		
2	1.84 (m), 1.68 (m)	27.4	1′′	4.36 (d, 6.7)	106.2
3	3.13 (dd, 11.6, 4.0)	90.0	2′′	3.88 (m)	75.5
4		40.5	3′′	3.85 (m)	83.2
5	0.77 (brd, 11.3)	57.6	4′′	4.04 (m)	70.8
6	1.53 (m), 1.39 (m)	19.5	5′′	3.83 (m), 3.53 (m)	66.7
7	1.47 (m), 1.31 (m)	34.1	Rha-(1→2)-Xyl		
8		40.9	1′′′	5.58 (brs)	101.3
9	1.57 (m)	49.1	2′′′	3.91 (m)	72.4
10		38.1	3′′′	3.69 (m)	72.3
11	2.24 (m), 1.91 (m)	24.7	4′′′	3.40 (m)	74.0
12	5.30 (t, 3.3)	124.7	5′′′	4.06 (m)	70.1
13		143.6	6′′′	1.21 (3H, d, 6.2)	18.0
14		43.1	Glc-(1→3)-Xyl		
15	1.75 (m), 1.11 (brd, 14.2)	29.1	1′′′′	4.64 (d, 7.1)	103.5
16	2.12 (td, 14.2, 3.3), 1.91 (m)	25.3	2′′′′	3.59 (m)	83.9
17		49.4	3′′′′	3.36 (m)	78.2
18	2.93 (dd, 13.7, 4.0)	42.2	4′′′′	3.35 (m)	71.3
19	1.92 (m), 1.31 (m)	47.7	5′′′′	3.58 (m)	78.4
20		36.4	6′′′′	3.84 (m), 3.66 (m)	62.6
21	4.81 (dd, 11.9, 4.7)	77.0	Glc-(1→2)-Glc-(1→3)-Xyl		
22	1.86 (m), 1.65 (m)	37.2	1′′′′′	4.74 (d, 7.2)	105.9
23	1.03 (3H, s)	28.7	2′′′′′	3.36 (m)	76.0
24	0.86 (3H, s)	17.5	3′′′′′	3.29 (m)	78.7
25	0.95 (3H, s)	16.3	4′′′′′	3.35 (m)	71.0
26	0.79 (3H, s)	17.8	5′′′′′	3.36 (m)	78.1
27	1.17 (3H, s)	26.3	6′′′′′	3.89 (m), 3.71 (m)	62.3
28		176.3	Glc (C-28)		
29	0.90 (3H, s)	29.3	1′′′′′′	5.38 (d, 8.2)	95.9
30	1.04 (3H, s)	18.9	2′′′′′′	3.30 (m)	74.0
1′		169.3	3′′′′′′	3.33 (m)	78.9
2′		129.0	4′′′′′′	3.35 (m)	71.1
3′	6.76 (td, 7.6, 1.2)	144.3	5′′′′′′	3.58 (m)	78.4
4′	2.24 (m), 1.83 (m)	24.4	6′′′′′′	3.80 (m), 3.67 (m)	62.4
5′	1.60 (2H, m)	41.7			
6′		73.7			
7′	5.91 (dd, 17.4, 10.8)	146.1			
8′	5.22 (dd, 17.4, 1.4)	112.6			
	5.05 (dd, 10.8, 1.4)				
9′	1.81 (3H, s)	12.6			
10′	1.27 (3H, s)	28.0			

*^a^* Recorded at 600 or 150 MHz for ^1^H and ^13^C, respectively.

**Table 2:** ^1^H- and ^13^C- NMR spectroscopic data of compounds **2**–**3***^a^* (in CD_3_OD).

Position	2		3	
	*δ*_H_ (mult, *J*, Hz)	*δ*_C_	*δ*_H_ (mult, *J*, Hz)	*δ*_C_
1		137.41		137.39
2	7.11 (d, 1.8)	112.92	7.11 (d, 1.9)	112.85
3		150.70		150.73
4		147.70		147.69
5	7.15 (d, 8.3)	117.66	7.15 (d, 8.3)	117.66
6	6.97 (dd, 8.3, 1.8)	121.19	6.96 (dd, 8.3, 1.9)	121.24
7	4.70 (d, 6.1)	77.87	4.70 (d, 6.1)	77.83
8	4.43 (dq, 6.2, 6.1)	81.55	4.43 (dq, 6.2, 6.1)	81.54
9	1.08 (d, 6.2)	16.53	1.09 (d, 6.3)	16.53
1′		134.07		134.07
2′	6.98 (d, 1.9)	111.10	6.99 (d, 1.9)	111.10
3′		151.96		151.96
4′		147.94		147.94
5′	6.90 (d, 8.3)	118.26	6.91 (d, 8.3)	118.25
6′	6.84 (dd, 8.3, 1.9)	120.13	6.84 (dd, 8.3, 1.9)	120.14
7′	6.33 (dd, 15.7, 1.6)	132.05	6.34 (dd, 15.7, 1.6)	132.05
8′	6.15 (dq, 15.7, 6.5)	125.02	6.15 (dq, 15.7, 6.6)	125.02
9′	1.85 (dd, 6.5, 1.6)	18.65	1.85 (dd, 6.6, 1.6)	18.65
3-OMe	3.87 (3H, s)	56.86	3.87 (3H, s)	56.86
3′-OMe	3.85 (3H, s)	56.66	3.85 (3H, s)	56.66
Glu				
1	4.88 (d, 7.5)	103.03	4.89 (d, 7.4)	103.00
2	3.48 (dd, 9.1, 7.5)	75.08	3.49 (dd, 9.1, 7.4)	75.08
3	3.45 (dd, 9.1, 8.5)	78.00	3.46 (dd, 9.1, 8.3)	78.01
4	3.40 (m)	71.51	3.40 (m)	71.51
5	3.40 (m)	78.36	3.40 (m)	78.37
6	3.86 (m)	62.67	3.86 (m)	62.67
	3.70 (dd, 12.1, 5.1)		3.69 (dd, 12.0, 5.1)	

*^a^* Recorded at 600 or 150 MHz for ^1^H and ^13^C, respectively.

## Supplementary Materials

The following are available online: ^1^H, ^13^C, 2D NMR, and HRMS spectra for compounds **1**–**3**.
