# Supplementary material for: Triterpenoid Saponin and Lignan Glycosides from the Traditional Medicine Elaeagnus angustifolia Flowers and Their Cytotoxic Activities
Source: Molecules. 2020 Jan 22;25(3):462. doi: 10.3390/molecules25030462 (PMC7037515; doi:10.3390/molecules25030462)

Triterpenoid Saponin and Lignan Glycosides from the Traditional Medicine *Elaeagnus angustifolia* Flowers and their Cytotoxic Activities

Jianxin Han,[1,2,†] Xiaoyu Chen,[2,†] Wei Liu,[2,3] Hao Cui,[3] and Tao Yuan[2,3,*]

[1]*Department of Food Science and Nutrition, School of Biosystems Engineering and Food Science, Zhejiang Key Laboratory for Agro-Food Processing, Zhejiang University, Hangzhou 310058, China; 77454287@qq.com (J.H.)*

[2]*The Key Laboratory of Plant Resources and Chemistry of Arid Zone, and State Key Laboratory of Xinjiang Indigenous Medicinal Plants Resource Utilization, Xinjiang Technical Institute of Physics and Chemistry, Chinese Academy of Sciences, Urumqi 830011, China; 492917544@qq.com (X.C.); 972088356@qq.com (W.L.)*

[3]*The Laboratory of Effective Substances of Jiangxi Genuine Medicinal Materials, College of Life Sciences, Jiangxi Normal University, Nanchang 330022, China; cuihaoeric@jxnu.edu.cn (H.C.)*

*Corresponding author: yuantao@ms.xjb.ac.cn (T.Y.)

[†]These authors contributed equally to this work.

Supporting Information

S1. ^{1}H NMR spectrum of terpengustifol A (**1**) in CD_3OD

S2. ^{13}C NMR spectrum of terpengustifol A (**1**) in CD_3OD

S3. HSQC spectrum of terpengustifol A (**1**) in CD_3OD

S4. ^{1}H-^{1}H COSY spectrum of terpengustifol A (**1**) in CD_3OD

S5. HMBC spectrum of terpengustifol A (**1**) in CD_3OD

S6. HMQC-TOCSY spectrum of terpengustifol A (**1**) in CD_3OD

S7. NOESY spectrum of terpengustifol A (**1**) in CD_3OD

S8. HRESIMS spectrum of terpengustifol A (**1**)

S9. ^1H NMR spectrum of the mixture of **2** and **3** in CD_3OD

S10. ^{13}C NMR spectrum of the mixture of **2** and **3** in CD_3OD

S11. ^1H-^1H COSY spectrum of the mixture of **2** and **3** in CD_3OD

S12. HSQC spectrum of the mixture of **2** and **3** in CD_3OD

S13. HMBC spectrum of the mixture of **2** and **3** in CD_3OD

S14. ^1H NMR spectrum of phengustifol A (**2**) in CD_3OD

S15. ^{13}C NMR spectrum of phengustifol A (**2**) in CD_3OD

S16. HRESIMS spectrum of phengustifol A (**2**)

S17. ^1H NMR spectrum of phengustifol B (**3**) in CD_3OD

S18. ^{13}C NMR spectrum of phengustifol B (**3**) in CD_3OD

S19. HRESIMS spectrum of phengustifol B (**3**)

S1. ^{1}H NMR spectrum of terpengustifol A (**1**) in CD_3OD

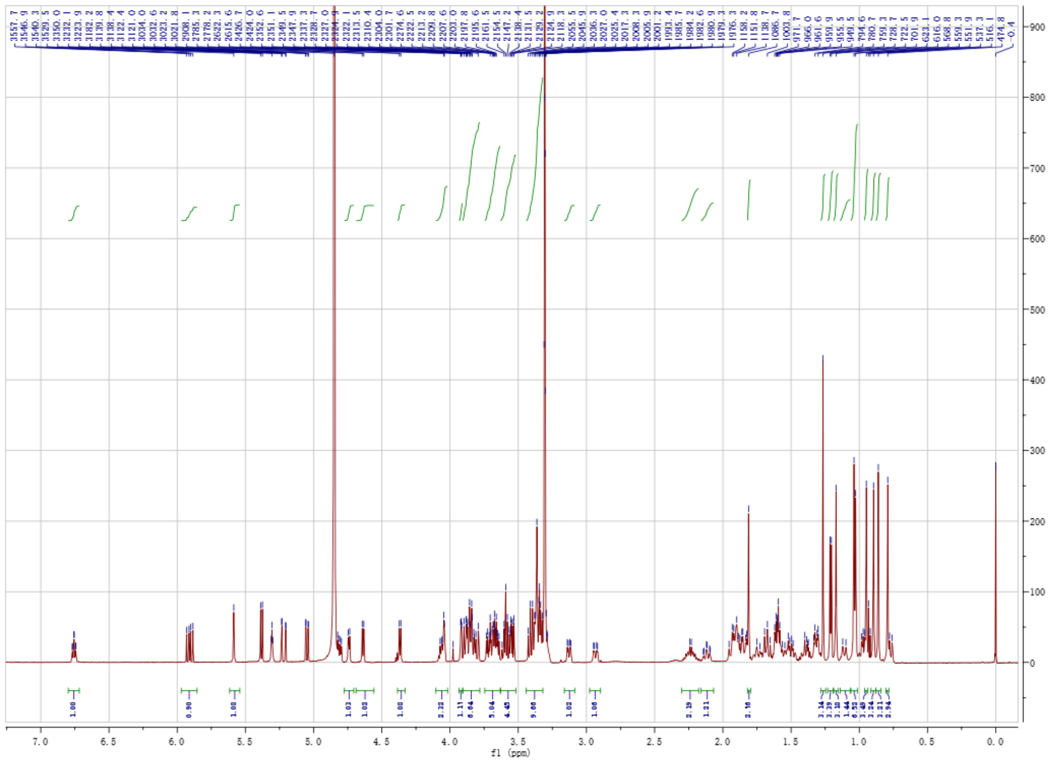

S2. ^{13}C NMR spectrum of terpengustifol A (**1**) in CD_3OD

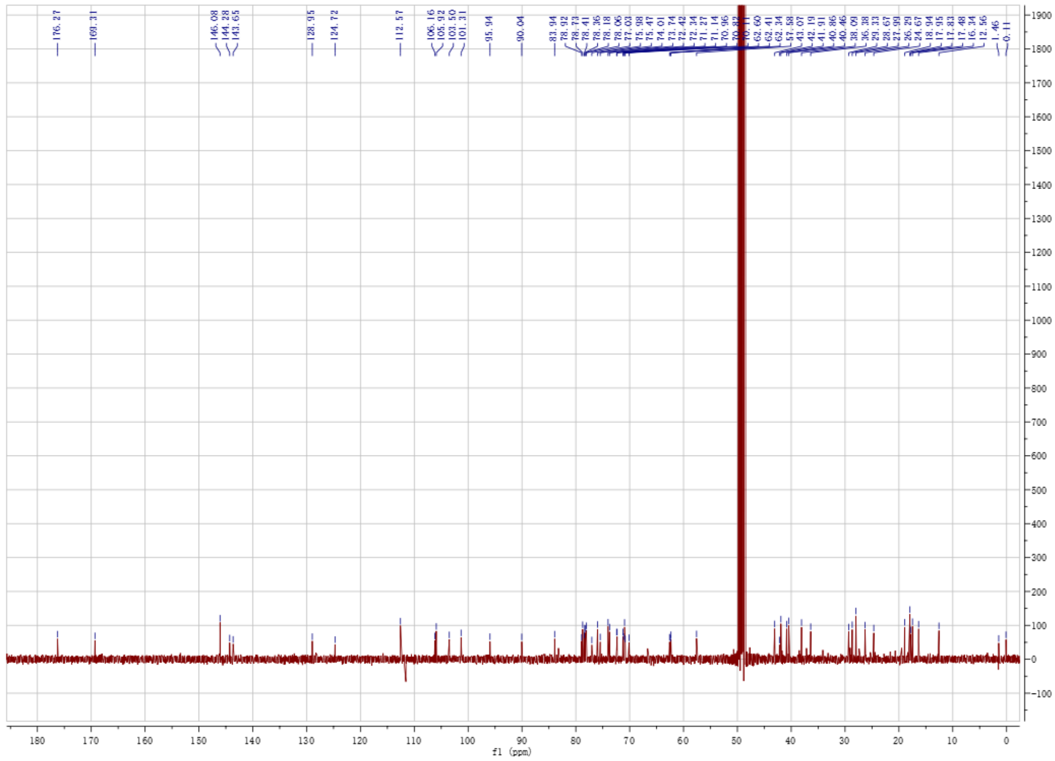

S3. HSQC spectrum of terpengustifol A (**1**) in CD_3OD

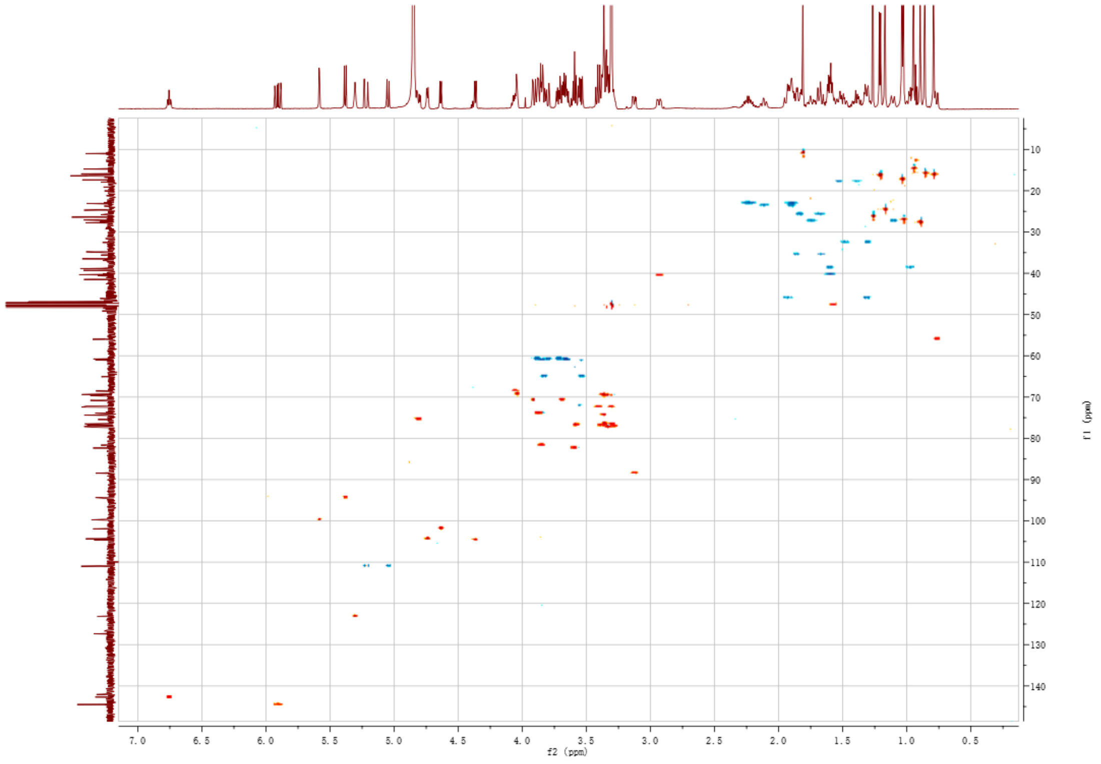

S4. ^1H-^1H COSY spectrum of terpengustifol A (**1**) in CD_3OD

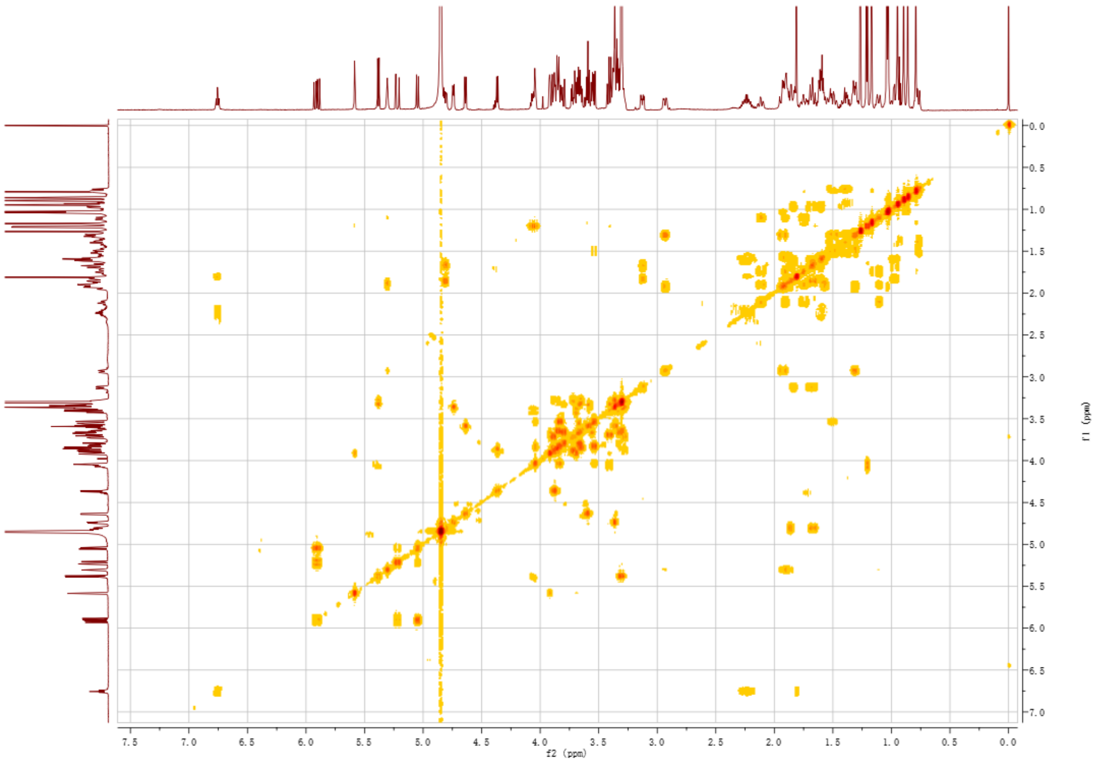

S5. HMBC spectrum of terpengustifol A (**1**) in CD_3OD

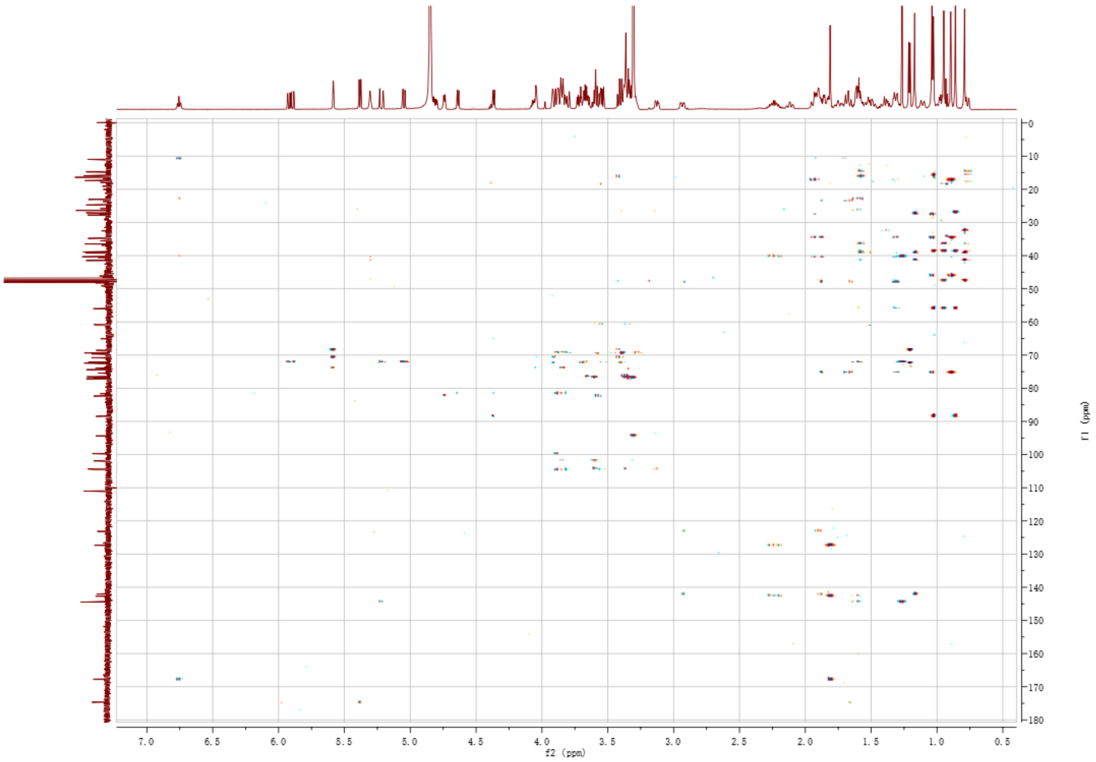

S6. HMQC-TOCSY spectrum of terpengustifol A (**1**) in CD_3OD

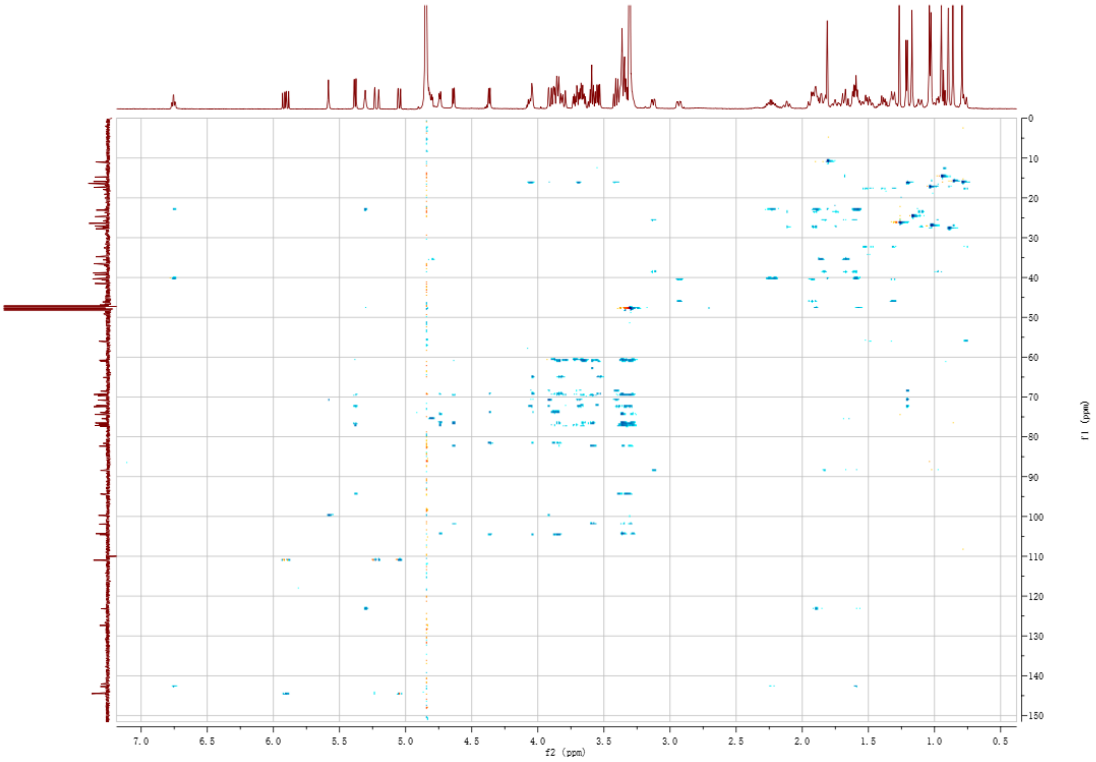

S7. NOESY spectrum of terpengustifol A (**1**) in CD_3OD

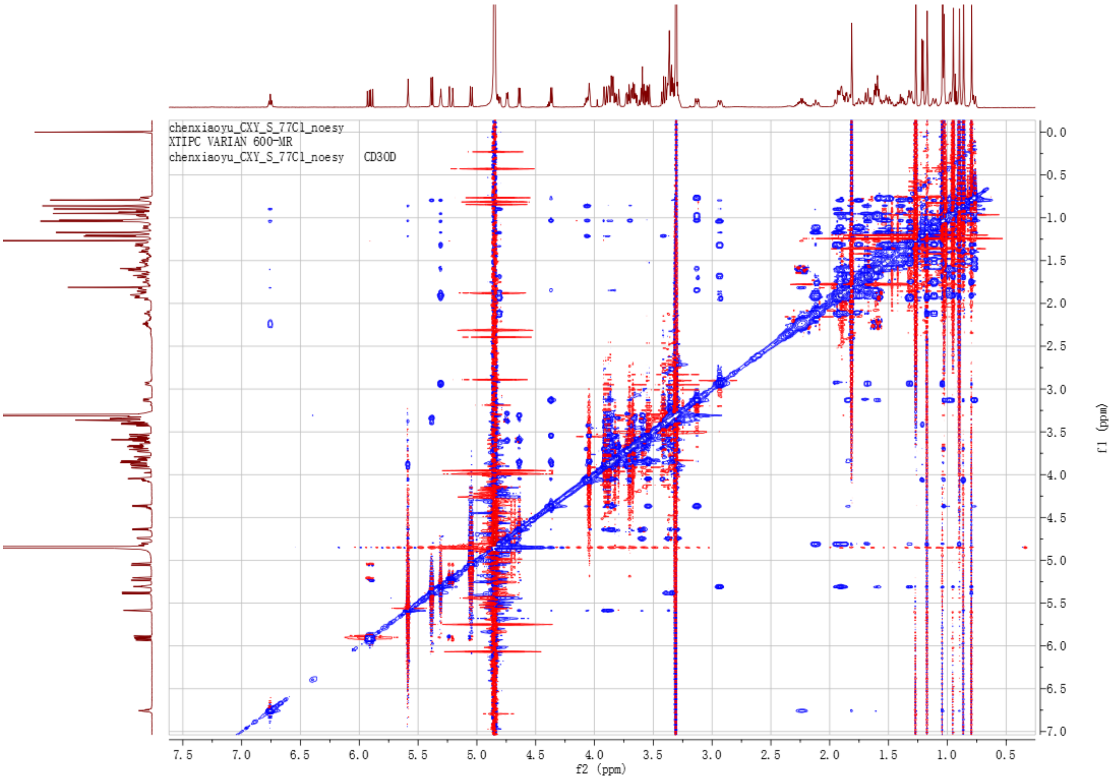

S8. HRESIMS spectrum of terpengustifol A (**1**)

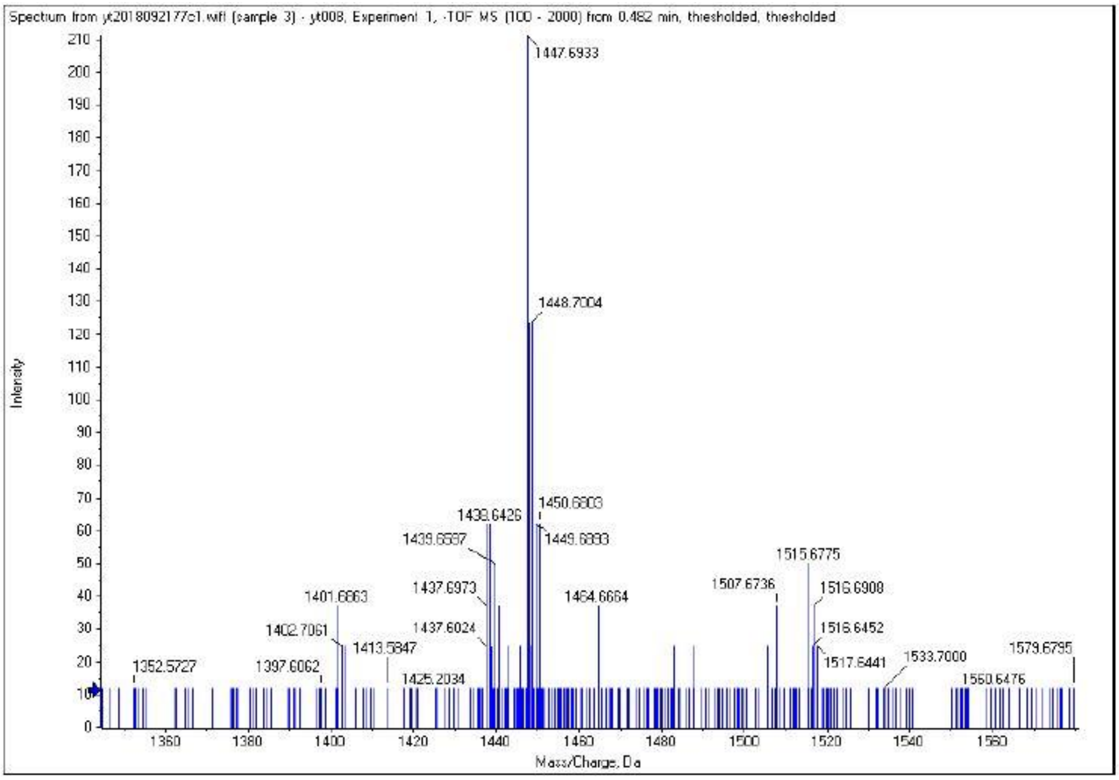

S9. ^{1}H NMR spectrum of the mixture of **2** and **3** in CD_3OD

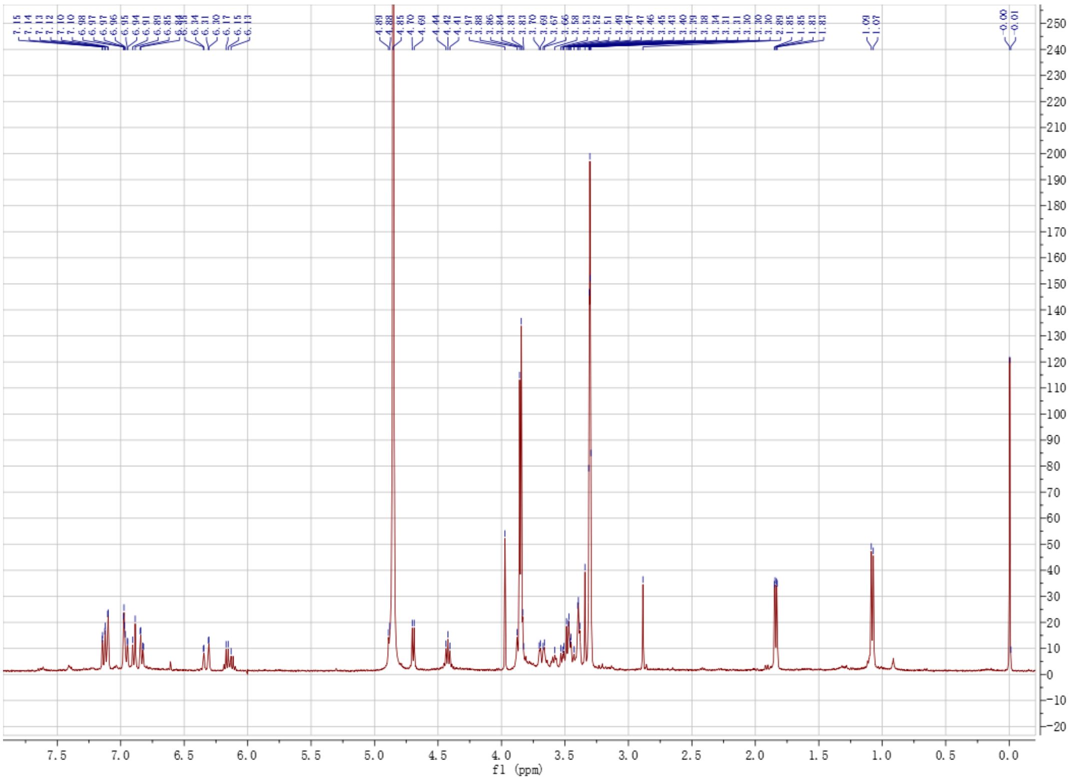

S10. ^{13}C NMR spectrum of the mixture of **2** and **3** in CD_3OD

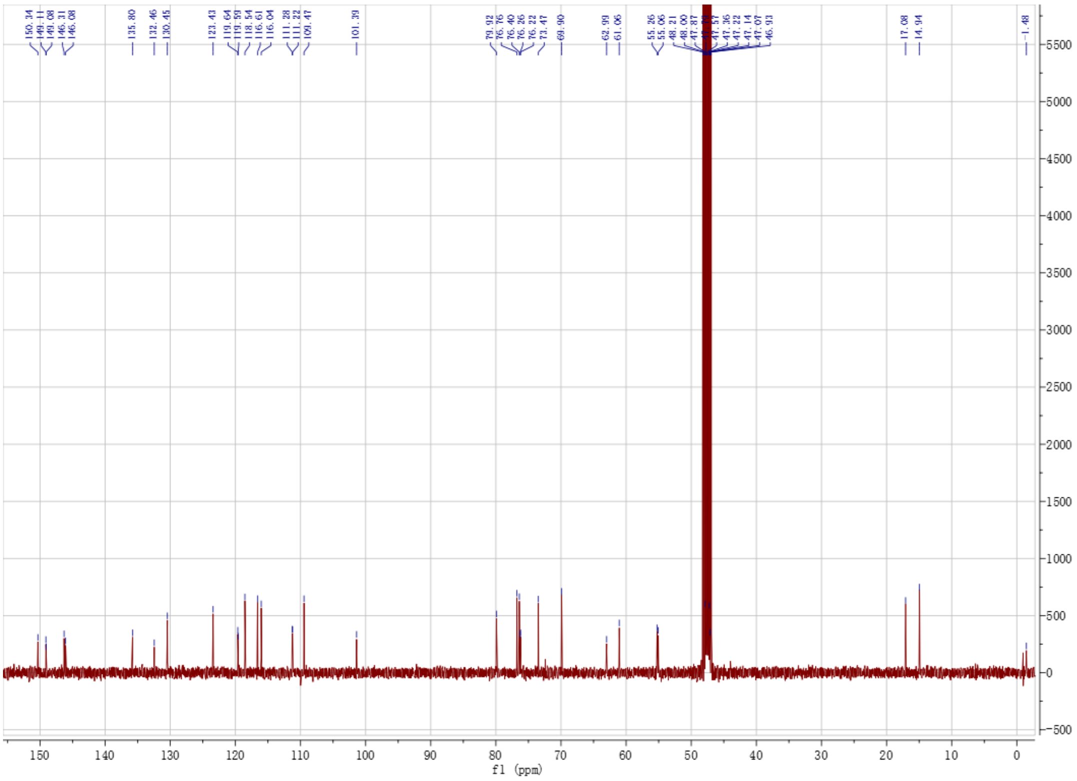

S11. ^{1}H-^{1}H COSY spectrum of the mixture of **2** and **3** in CD_3OD

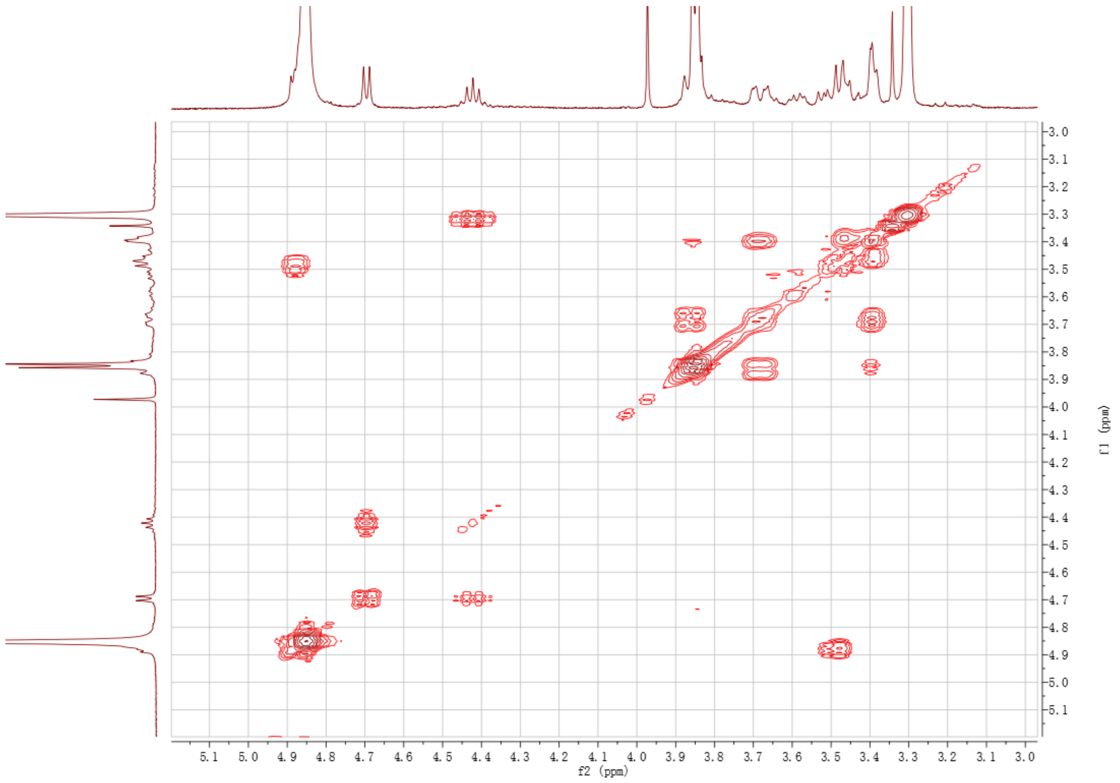

S12. HSQC spectrum of the mixture of **2** and **3** in CD_3OD

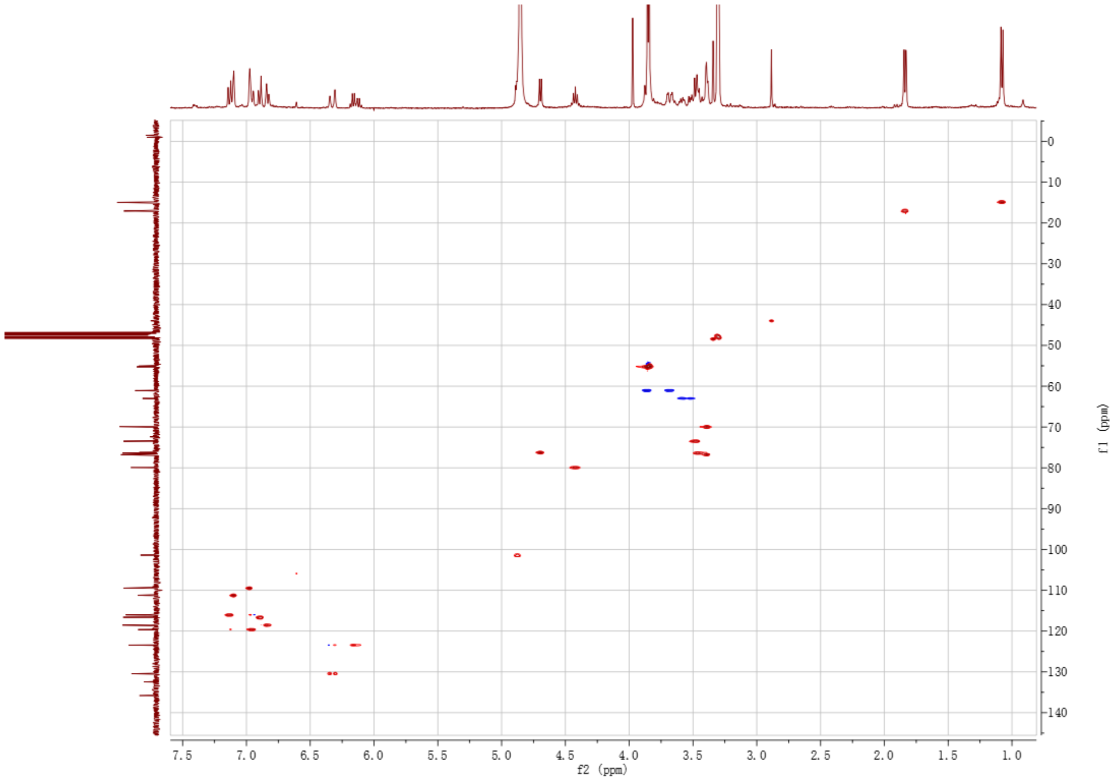

S13. HMBC spectrum of the mixture of **2** and **3** in CD_3OD

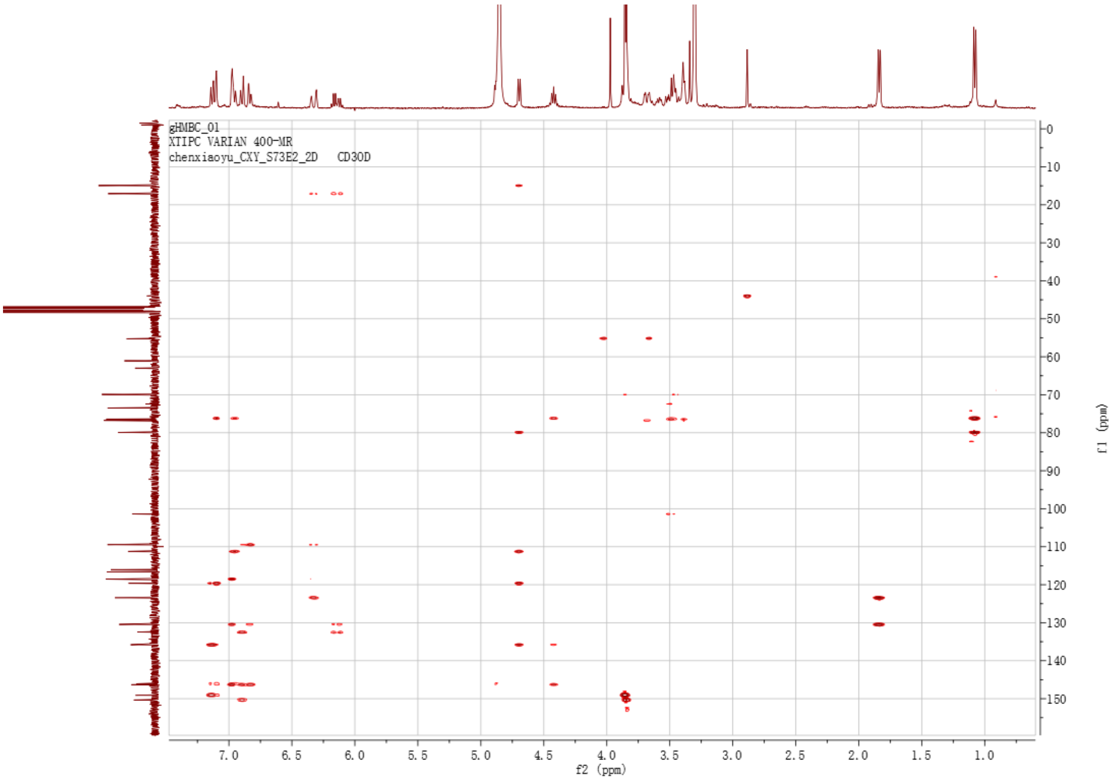

S14. ^{1}H NMR spectrum of phengustifol A (**2**) in CD_3OD

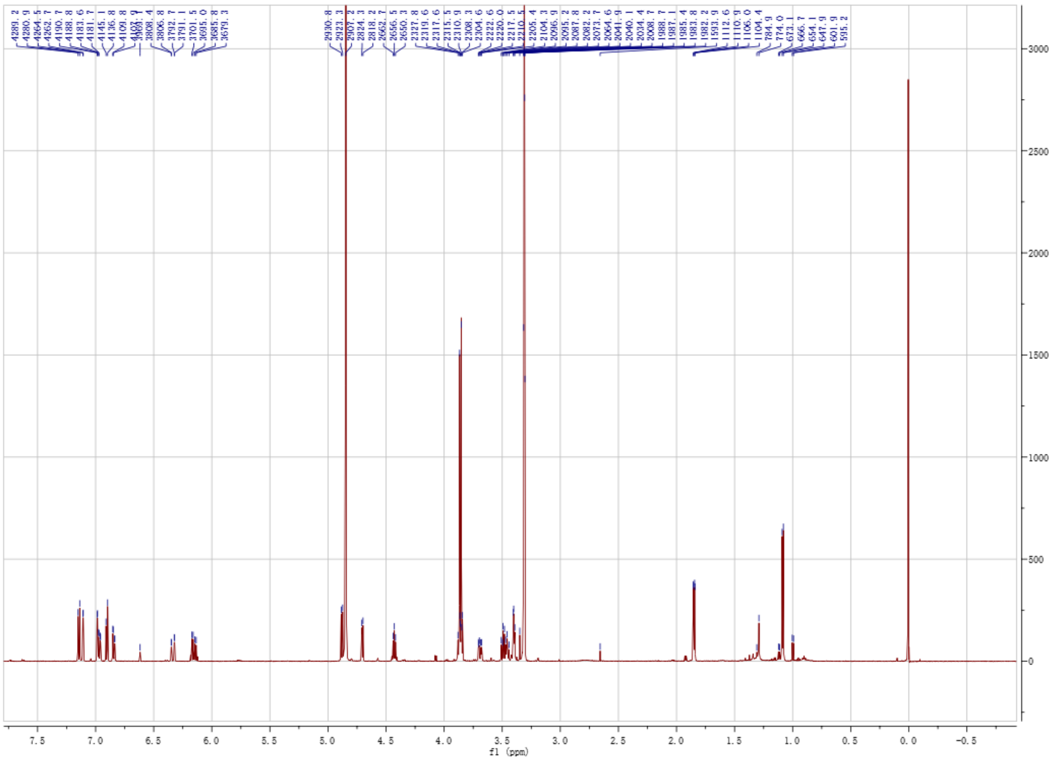

S15. ^{13}C NMR spectrum of phengustifol A (**2**) in CD_3OD

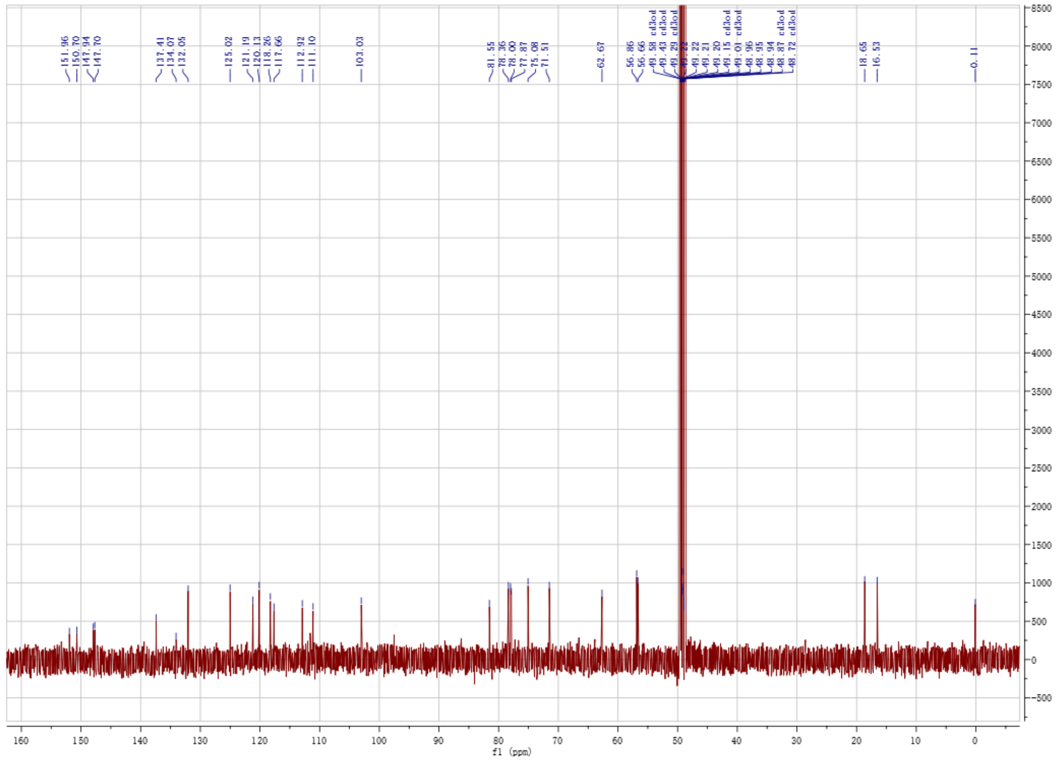

S16. HRESIMS spectrum of phengustifol A (**2**)

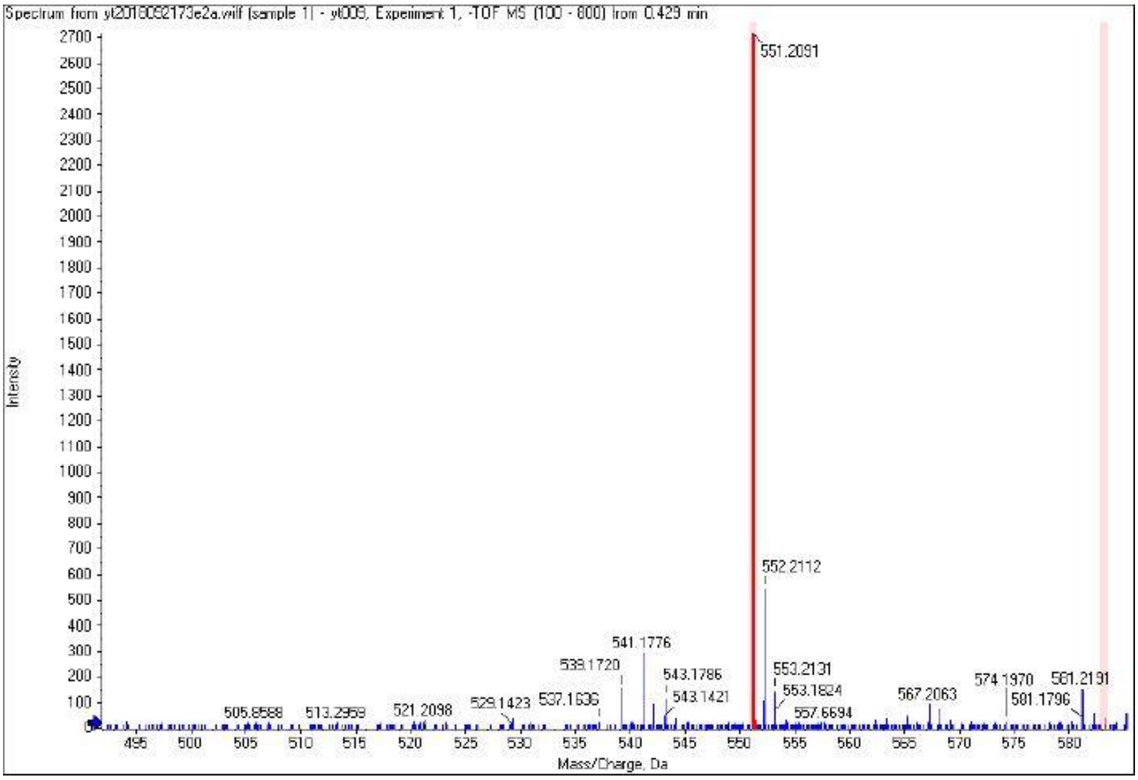

S17. ^{1}H NMR spectrum of phengustifol B (**3**) in CD_3OD

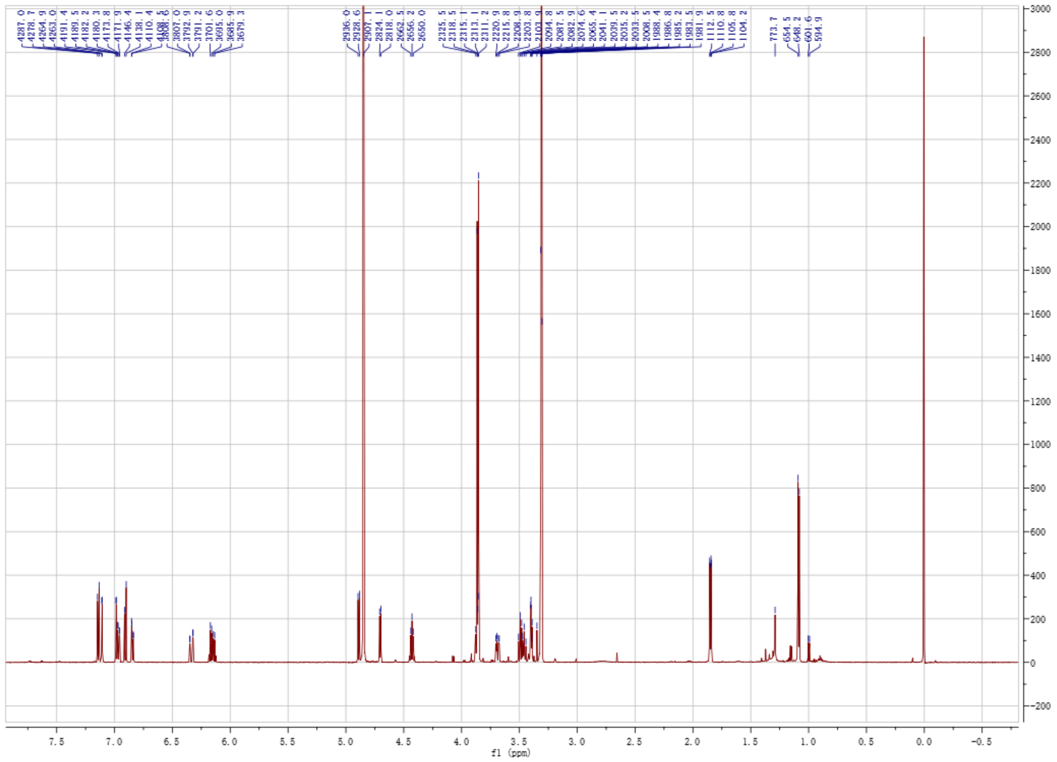

S18. ^{13}C NMR spectrum of phengustifol B (**3**) in CD_3OD

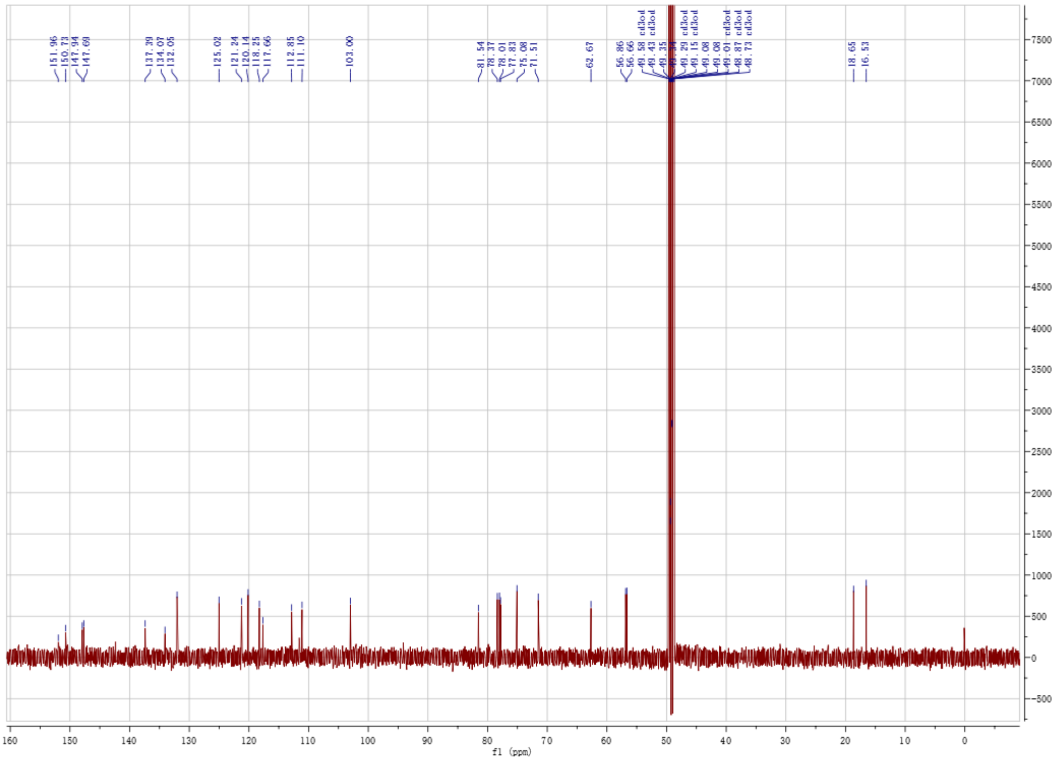

S19. HRESIMS spectrum of phengustifol B (**3**)

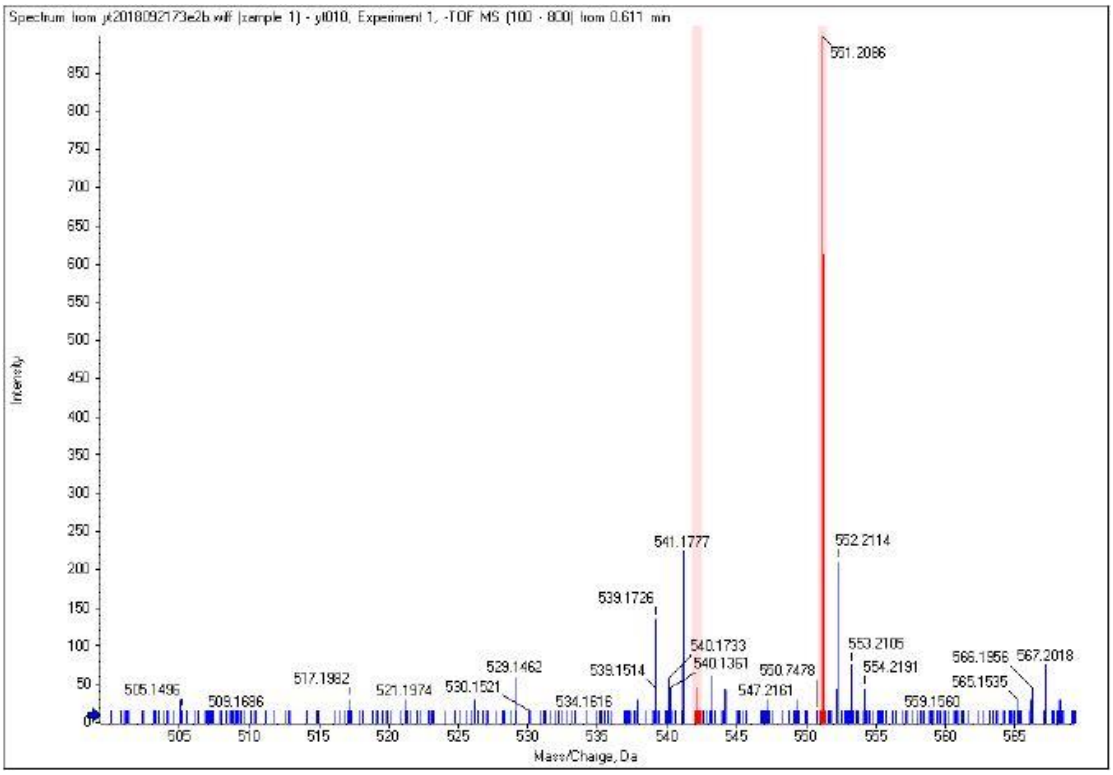

Supplement: Supplementary file 1 [file molecules-25-00462-s001.pdf]
